# Supplementary material for: A Humanized Yeast Phenomic Model of Deoxycytidine Kinase to Predict Genetic Buffering of Nucleoside Analog Cytotoxicity
Source: Genes (Basel). 2019 Sep 30;10(10):770. doi: 10.3390/genes10100770 (PMC6826991; doi:10.3390/genes10100770)
Supplement: Supplementary file 1 [file genes-10-00770-s001.zip › Additional_Files/Additional_File7_GOTermHeatmaps.zip!/Additional_File7_GOTermHeatmaps/regulation_of_DNA-templated_transcription,_elongation.pdf]

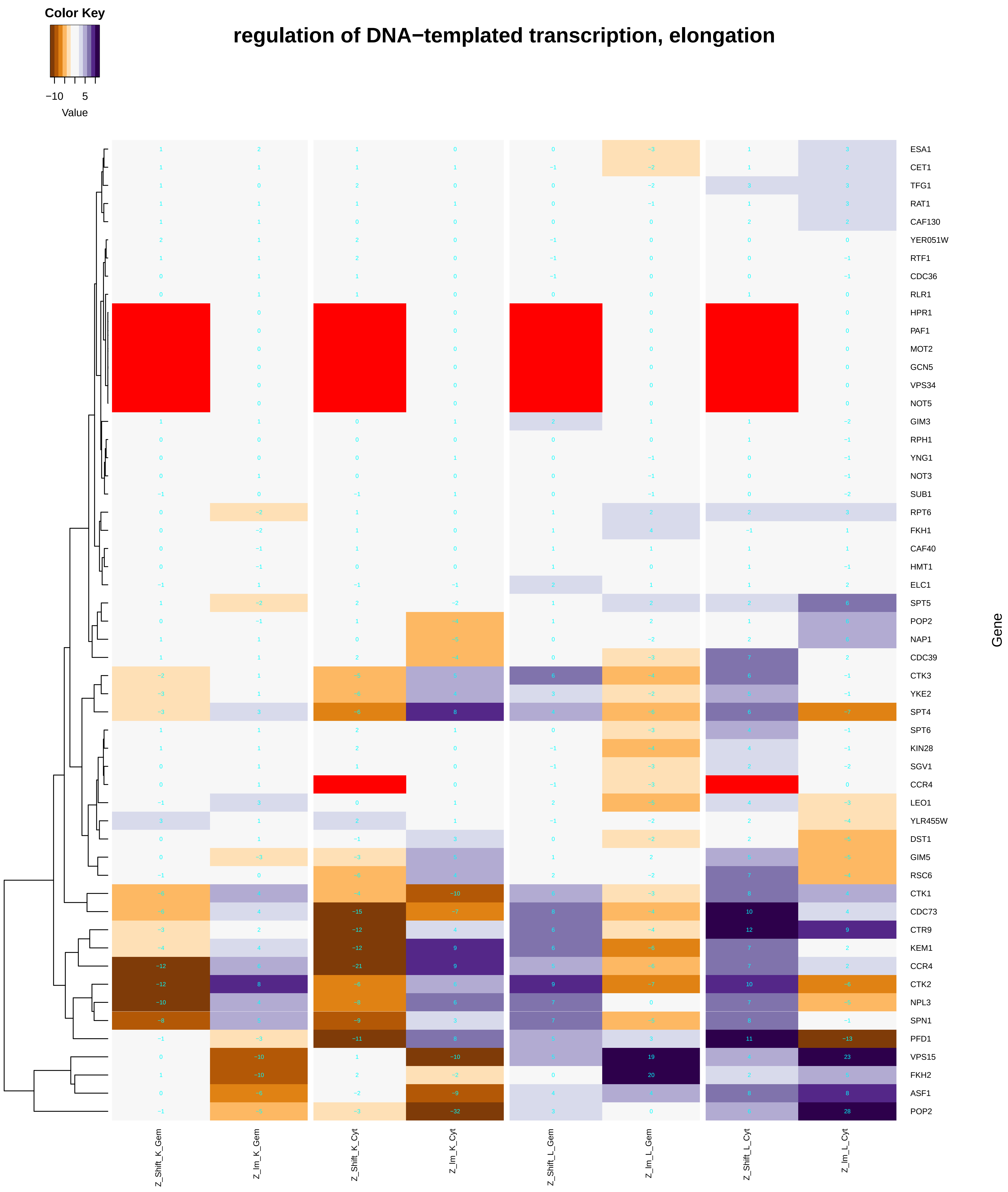
regulation of DNA−templated transcription, elongation
Color Key
−10
5
Value
ESA1
CET1
TFG1
RAT1
CAF130
YER051W
RTF1
CDC36
RLR1
HPR1
PAF1
MOT2
GCN5
VPS34
NOT5
GIM3
RPH1
YNG1
NOT3
SUB1
RPT6
FKH1
CAF40
HMT1
ELC1
SPT5
POP2
NAP1
CDC39
CTK3
YKE2
SPT4
SPT6
KIN28
SGV1
CCR4
LEO1
YLR455W
DST1
GIM5
RSC6
CTK1
CDC73
CTR9
KEM1
CCR4
CTK2
NPL3
SPN1
PFD1
VPS15
FKH2
ASF1
POP2
Gene
Z_Shift_K_Gem
Z_Im_K_Gem
Z_Shift_K_Cyt
Z_Im_K_Cyt
Z_Shift_L_Gem
Z_Im_L_Gem
Z_Shift_L_Cyt
Z_Im_L_Cyt

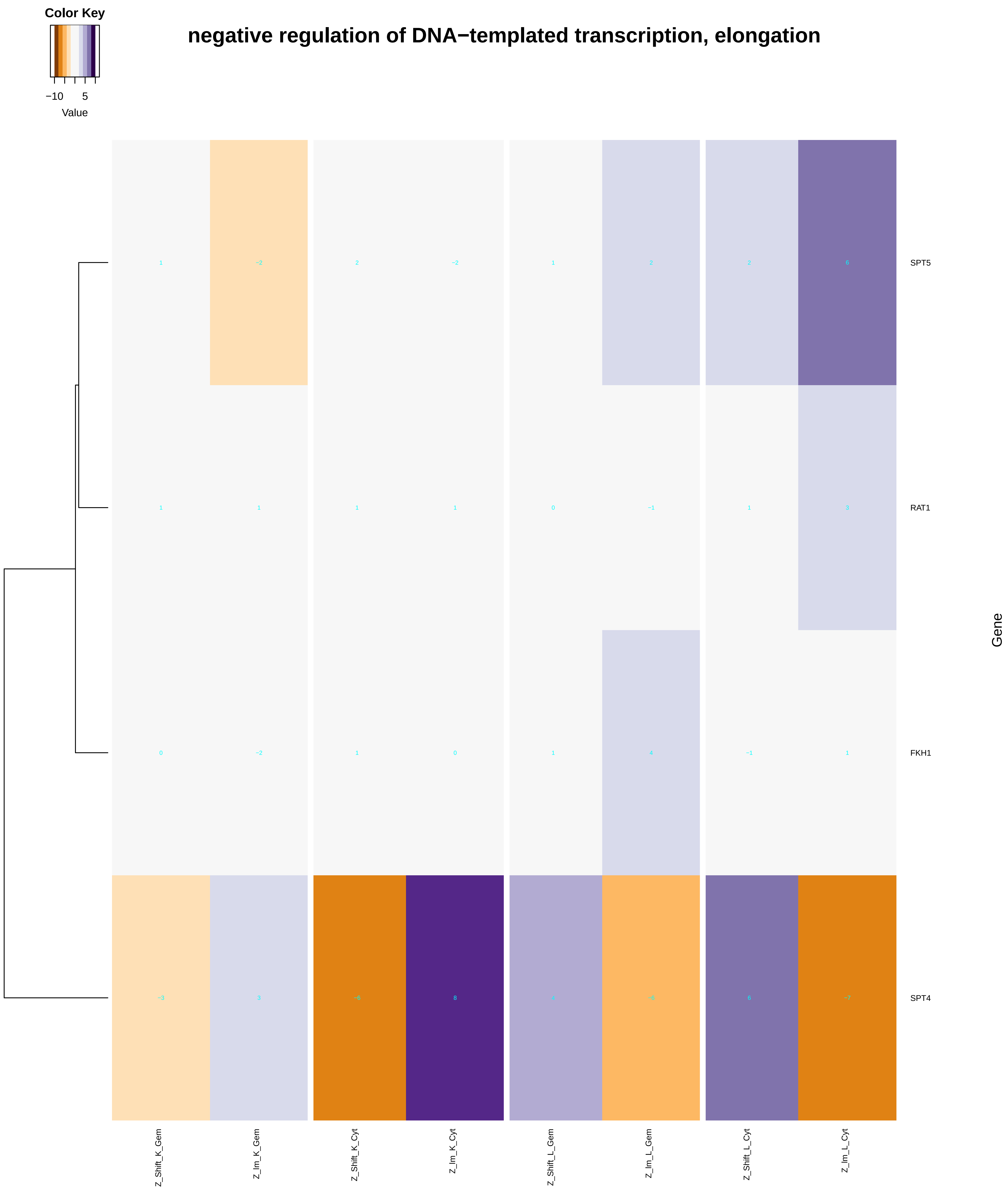

Color Key
−10
5
Value
negative regulation of DNA−templated transcription, elongation
SPT5
RAT1
FKH1
SPT4
Gene
Z_Shift_K_Gem
Z_Im_K_Gem
Z_Shift_K_Cyt
Z_Im_K_Cyt
Z_Shift_L_Gem
Z_Im_L_Gem
Z_Shift_L_Cyt
Z_Im_L_Cyt

positive regulation of DNA−templated transcription, elongation

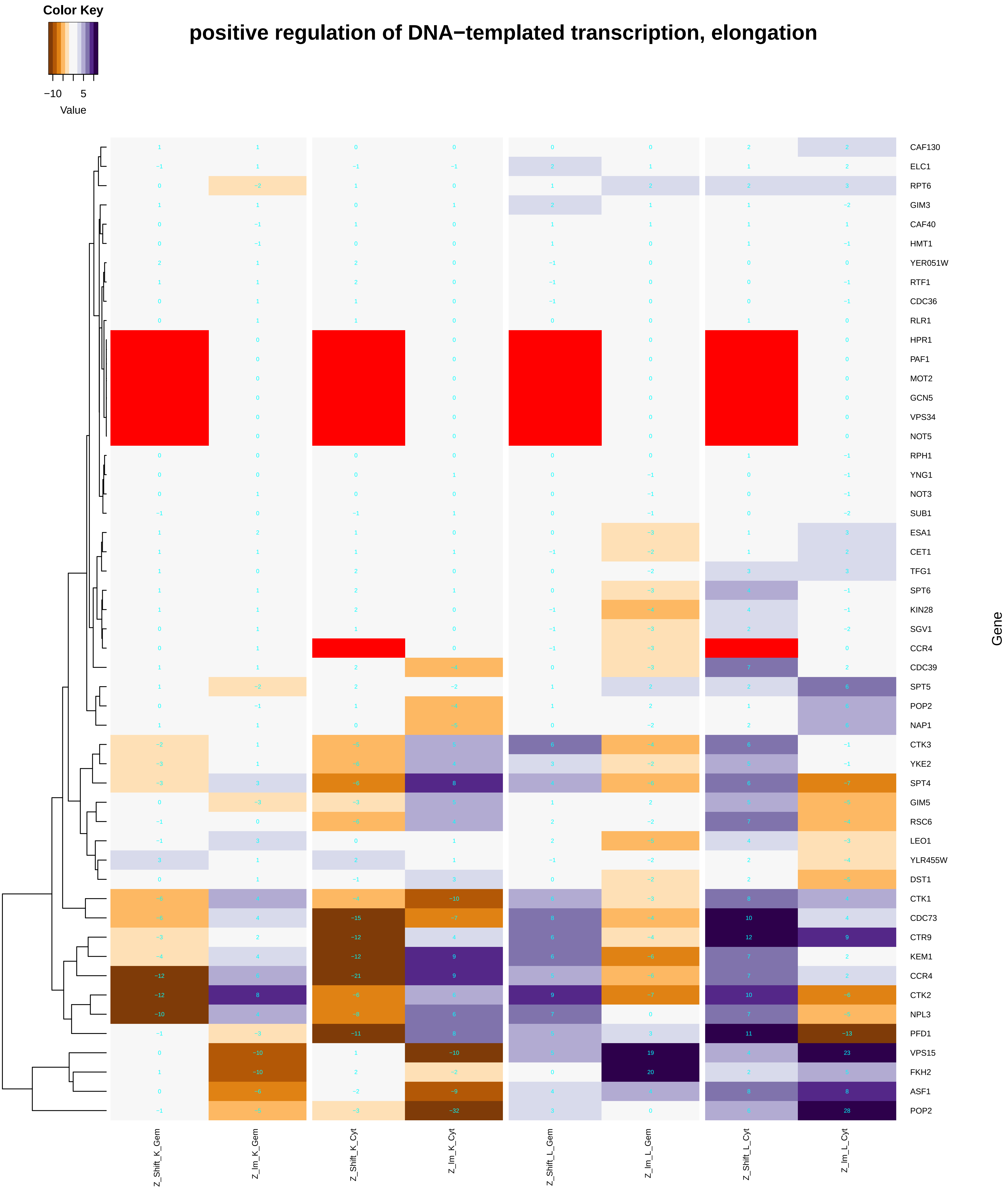

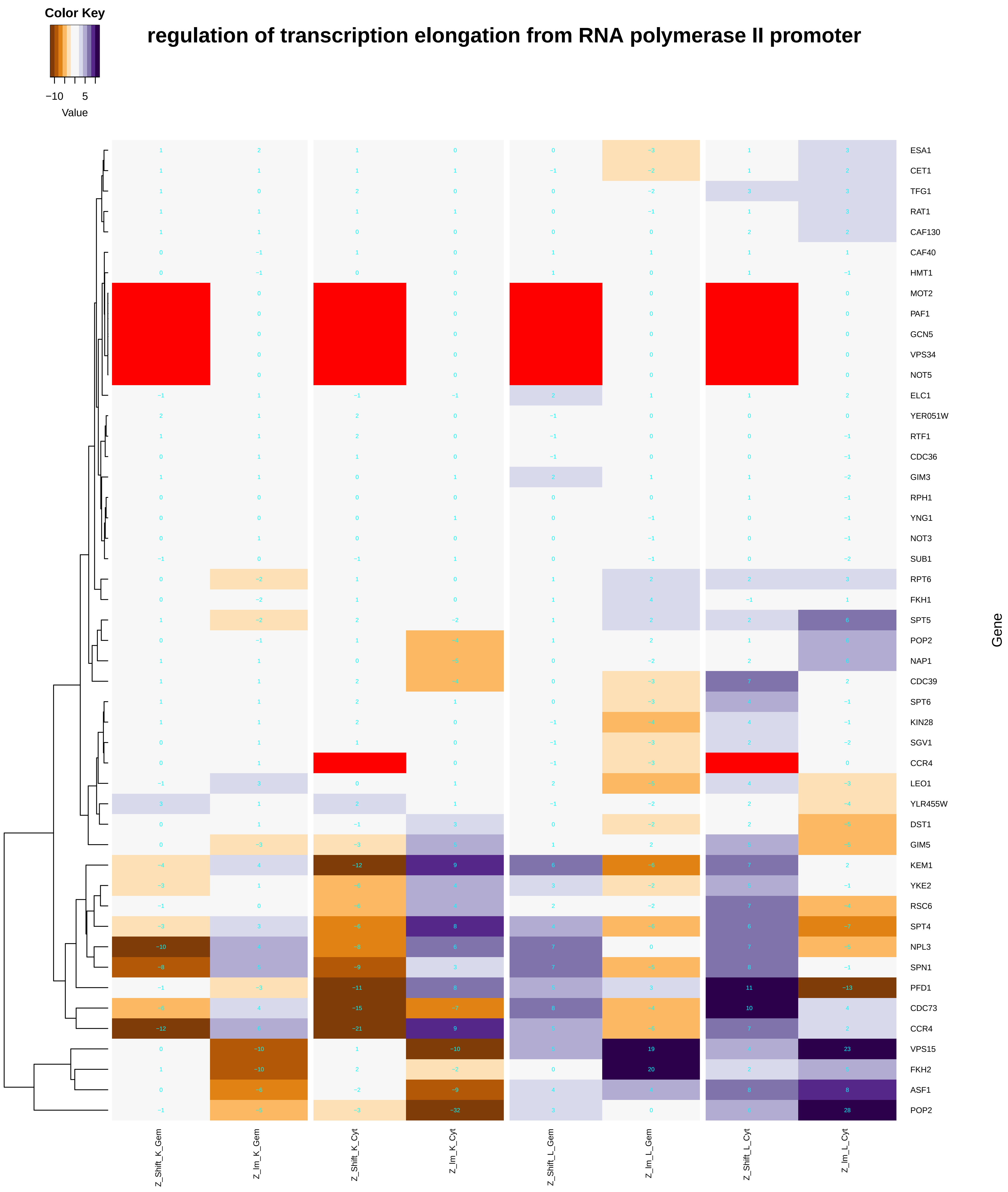
regulation of transcription elongation from RNA polymerase II promoter
Color Key
−10
5
Value
Gene
ESA1
CET1
TFG1
RAT1
CAF130
CAF40
HMT1
MOT2
PAF1
GCN5
VPS34
NOT5
ELC1
YER051W
RTF1
CDC36
GIM3
RPH1
YNG1
NOT3
SUB1
RPT6
FKH1
SPT5
POP2
NAP1
CDC39
SPT6
KIN28
SGV1
CCR4
LEO1
YLR455W
DST1
GIM5
KEM1
YKE2
RSC6
SPT4
NPL3
SPN1
PFD1
CDC73
CCR4
VPS15
FKH2
ASF1
POP2
Z_Shift_K_Gem
Z_Im_K_Gem
Z_Shift_K_Cyt
Z_Im_K_Cyt
Z_Shift_L_Gem
Z_Im_L_Gem
Z_Shift_L_Cyt
Z_Im_L_Cyt

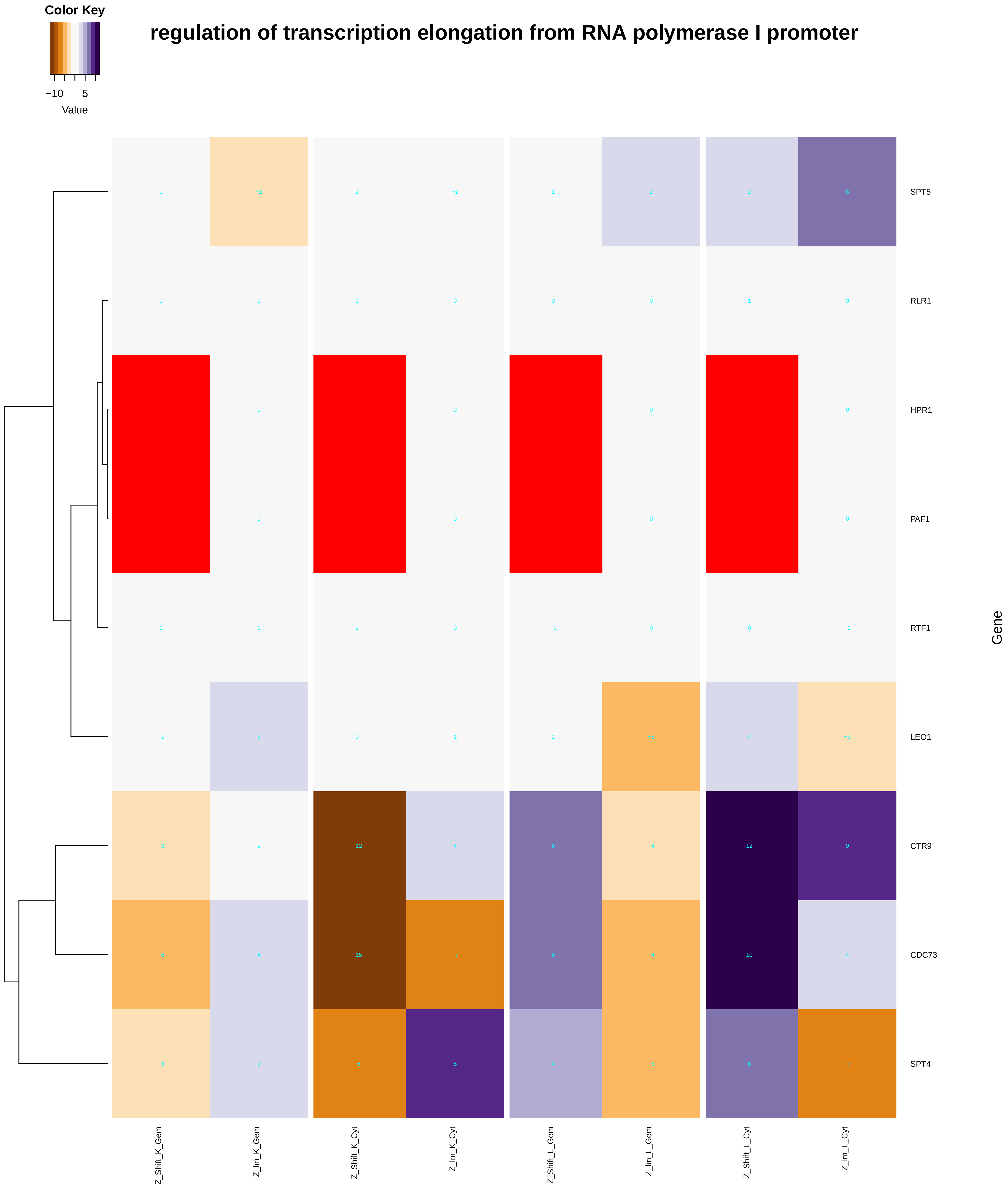
regulation of transcription elongation from RNA polymerase I promoter
Color Key
−10
5
Value
SPT5
RLR1
HPR1
PAF1
RTF1
LEO1
CTR9
CDC73
SPT4
Gene
Z_Shift_K_Gem
Z_Im_K_Gem
Z_Shift_K_Cyt
Z_Im_K_Cyt
Z_Shift_L_Gem
Z_Im_L_Gem
Z_Shift_L_Cyt
Z_Im_L_Cyt

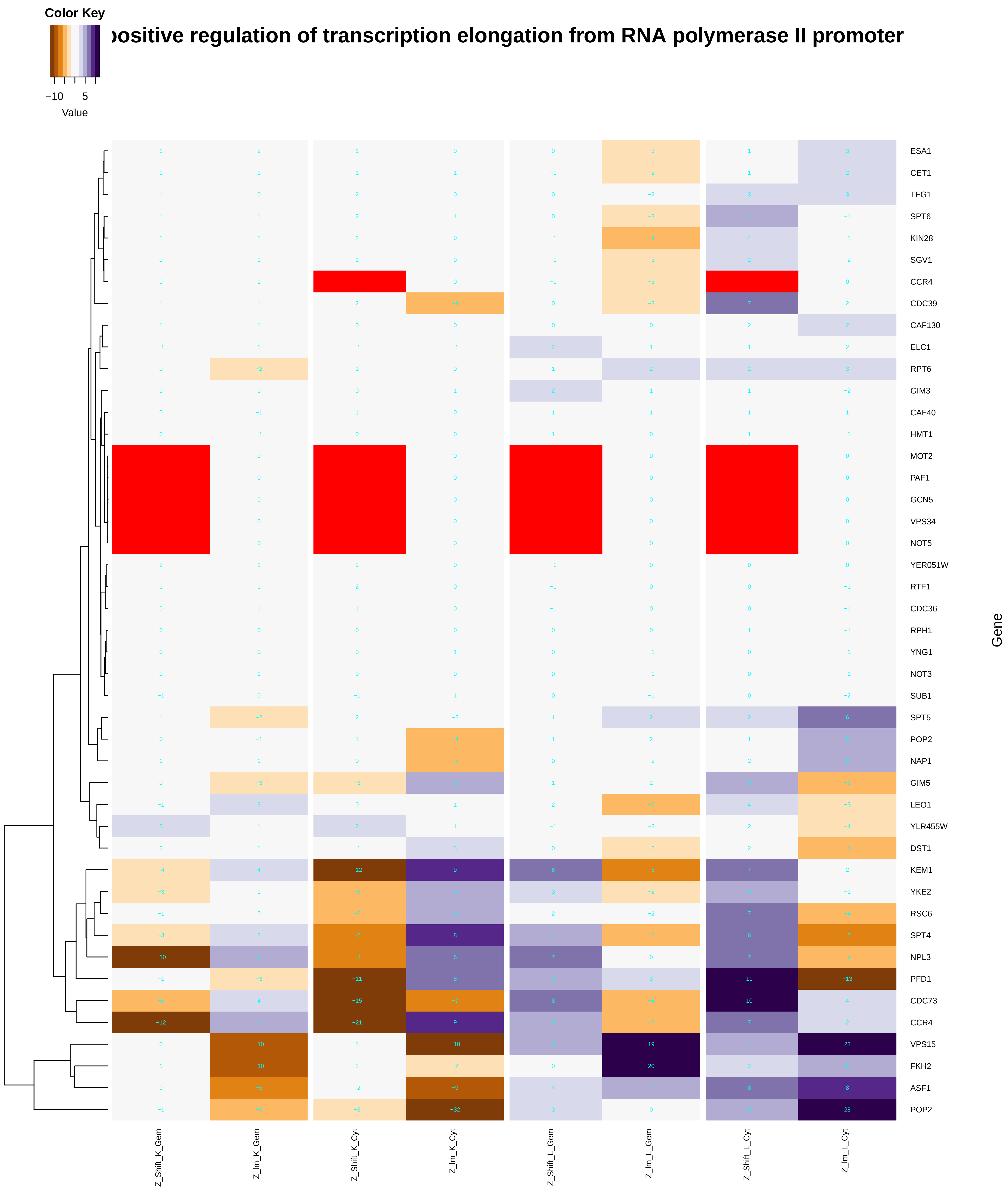
Color Key
−10
5
Value
ositive regulation of transcription elongation from RNA polymerase II promoter
ESA1
CET1
TFG1
SPT6
KIN28
SGV1
CCR4
CDC39
CAF130
ELC1
RPT6
GIM3
CAF40
HMT1
MOT2
PAF1
GCN5
VPS34
NOT5
YER051W
RTF1
CDC36
RPH1
YNG1
NOT3
SUB1
SPT5
POP2
NAP1
GIM5
LEO1
YLR455W
DST1
KEM1
YKE2
RSC6
SPT4
NPL3
PFD1
CDC73
CCR4
VPS15
FKH2
ASF1
POP2
Gene
Z_Shift_K_Gem
Z_lm_K_Gem
Z_Shift_K_Cyt
Z_lm_K_Cyt
Z_Shift_L_Gem
Z_lm_L_Gem
Z_Shift_L_Cyt
Z_lm_L_Cyt

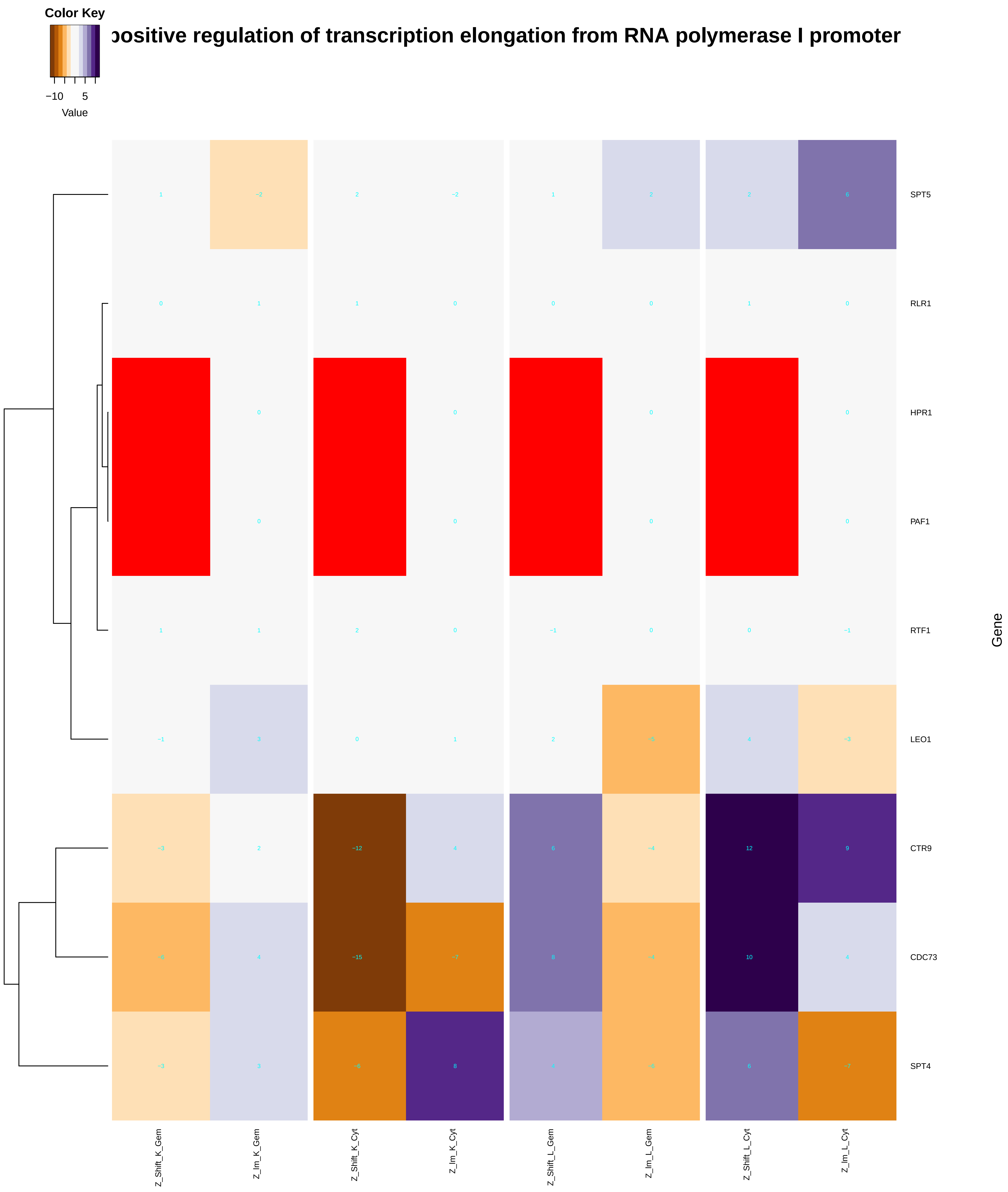

Color Key
−10
5
Value
ositive regulation of transcription elongation from RNA polymerase I promoter
SPT5
RLR1
HPR1
PAF1
RTF1
LEO1
CTR9
CDC73
SPT4
Gene
Z_Shift_K_Gem
Z_Im_K_Gem
Z_Shift_K_Cyt
Z_Im_K_Cyt
Z_Shift_L_Gem
Z_Im_L_Gem
Z_Shift_L_Cyt
Z_Im_L_Cyt
